# A Rolling-Horizon Lane-Level Queue Prediction System: Algorithm Validation and Field Deployment in a Cooperative Intelligent Transport System (C-ITS) Testbed

**DOI:** 10.3390/s26216989

**Published:** 2026-11-05

**Authors:** Inchul Yang, Hyoungsoo Kim, Junhyung Lee

**Affiliations:** Korea Institute of Civil Engineering and Building Technology (KICT), 283 Goyang-daero, Ilsanseo-gu, Goyang-si 10223, Gyeonggi-do, Republic of Korea; hsookim@kict.re.kr (H.K.); jun@kict.re.kr (J.L.)

**Keywords:** connected and autonomous vehicles, lane-level queue length prediction, cooperative intelligent transport systems, shockwave theory, platoon dispersion model, SAE J2735

## Abstract

**Highlights:**

**What are the main findings?**

• LQS predicts lane-level queue length in real time from upstream movement counts and signal phase, combining a platoon dispersion model with a shockwave-theory queue model on a rolling-horizon scheme and using only information available at prediction time.
• On held-out validation cycles, the mean cycle-maximum relative error on the four signal-controlled lanes is 24.5%—lower than equal-split, running-mean lane-share, and point-queue baselines (33.2–52.8%), and equal to an oracle that knows the current cycle’s lane use.

**What are the implications of the main findings?**

• A standards-based SAE J2735 message extension makes lane-level queue predictions usable by connected and autonomous vehicles for lane-selection and lane-change decisions.
• The two empirically calibrated parameters are coupled and the departure-wave speed lies close to an instability threshold, so both should be re-calibrated jointly at a new site; right-turn lanes that discharge on red require a different model.

**Abstract:**

Lane selection and lane-change decisions by connected and autonomous vehicles (CAVs) require queue-length information at lane resolution, which corridor-level estimation methods do not provide. This paper presents the Lane-by-lane Queue Information Prediction System (LQS), which predicts per-lane queue length from upstream movement-traffic volume and signal-phase data by combining a platoon dispersion model with a shockwave-theory queue model on a rolling-horizon scheme. We describe its integration into a live C-ITS stack through an SAE J2735 message extension and its deployment at a testbed in Hwaseong, Republic of Korea, where processing latency was measured (313.6 ms average) but field accuracy was not. In an independent microsimulation testbed (Goyang, Republic of Korea), with parameters calibrated on signal cycles 6–15 and evaluated on held-out cycles 16–21, the mean cycle-maximum relative error on the four signal-controlled lanes is 24.5% (17.1–30.0%), against 33.2–52.8% for baseline methods; the right-turn lane, which discharges on red, is reported separately (51.5%). Because cycle-maximum and full-series errors rank lanes differently, we also report on RMSE normalized by mean queue, bias, and correlation, which reconcile the two metrics. An ablation shows that the two empirically tuned parameters are coupled.

## 1. Introduction

### 1.1. Motivation and Problem Statement

Traffic queues at signalized intersections do not distribute evenly across lanes: adjacent lanes serving different turning movements, or the same movement under uneven driver lane choice, routinely carry substantially different queue lengths at the same moment in time. For a human-driven vehicle this imbalance is a minor inconvenience; for a connected or autonomous vehicle (CAV) making an automated lane-choice or lane-change decision on its approach to an intersection, it is information the vehicle’s own sensors often cannot supply in time. A CAV traveling straight through an intersection with a long queue ahead in its current lane, but a short queue in an adjacent lane serving the same movement, benefits directly from being told which lane is shorter before it is close enough to observe this itself—particularly when the queue is around a curve, beyond sensor range, or forming faster than onboard perception can track. Similarly, a vehicle approaching a mandatory lane change (for example, merging out of a lane that will become a right-turn-only pocket) needs enough advance warning on the target lane’s queue state to execute the change safely rather than abruptly.

These are not hypothetical scenarios but concrete use cases documented in the engineering studies behind this system: avoiding left-turn-pocket queue overflow by re-routing straight-through traffic to a through lane before the pocket backs up; choosing the shorter of several lanes serving the same downstream movement to reduce travel time; timing a mandatory lane change relative to the remaining safe distance to a queue’s tail; and determining the optimal point at which a following vehicle should begin braking for a queue that is not yet visible to its own sensors. Addressing these needs requires queue-length information at lane-level granularity, delivered with enough lead time to act on— which in turn requires predicting near-future queue length, not merely observing its current state, since a vehicle several hundred meters upstream needs to know what the queue will look like when it arrives, not what it looked like when the data was collected. This is the problem the Lane-by-lane Queue Information Prediction System (LQS) is designed to solve.

Infrastructure-supplied queue information also complements the vehicle’s own perception. Onboard sensors observe only what lies within their line of sight and range, and at signalized intersections, the tail of a queue in an adjacent lane, or the full extent of a queue ahead of a large vehicle, is frequently occluded. Cooperative perception and shared situational awareness address this limitation by fusing observations from infrastructure sensors or other vehicles, delivered over V2X communication, with the ego vehicle’s own sensing [1,2]. Distributed set-membership estimation has been used in the same spirit to keep the ego vehicle’s estimates of surrounding vehicles’ states bounded under unknown inputs and unknown-but-bounded noise, and to support collision warning built on those estimates [3]. LQS can be viewed as a traffic-state counterpart to these object-level approaches: rather than sharing detected objects, it shares a predicted lane-level queue state, computed from infrastructure-side detection, over a horizon that extends beyond what the ego vehicle can currently observe.

### 1.2. Related Work

Queue-length estimation and prediction at signalized intersections is a long-studied and now very extensively populated research area, and the literature is more naturally organized by the sensing and estimation paradigm that each strand relies on than by any single unifying theory. The first, classical paradigm estimates queue length from fixed infrastructure sensors—inductive loop detectors chief among them—combined with shockwave or point-queue traffic-flow theory; Liu et al.’s method for congested signalized intersections is a foundational example of this paradigm and remains a standard point of comparison for later work [4].

The second, now considerably larger paradigm exploits vehicle-to-infrastructure or probe-vehicle data—GPS trajectories, dedicated short-range-communication beacons, or range-measurement sensors reporting a subset of vehicles’ positions—to estimate queue length without relying on fixed detectors at all. Within this paradigm, one active line of work derives estimators directly from individual probe-vehicle trajectories or their arrival/departure timing, either analytically or via a shockwave-consistent reconstruction of the queue profile [5,6,7,8], while a second line instead treats the estimation problem as a filtering or learning problem, using Kalman-filter or shockwave-neural-network formulations to fuse sparse probe observations across signal cycles [9,10,11]. A recurring finding across this literature, directly relevant to the algorithm validated in this paper, is that estimation accuracy is highly sensitive to connected/probe-vehicle market penetration rate, particularly in the low-penetration regime closest to the current field conditions [10,12]. Several recent studies have extended this paradigm from a research setting toward an operational one, evaluating connected-vehicle-based queue estimation as an input to real-time signal control or vehicle-to-infrastructure message pipelines rather than as an offline estimator alone [13,14]—the same operational framing this paper adopts for its own field deployment (Section 2.9).

A third strand addresses queue-length estimation specifically at lane-level, rather than an approach- or corridor-level resolution—the resolution this paper’s own contribution targets. Lane-level estimates have been obtained from automatic-vehicle-identification and advance-detector data linking a vehicle’s detections across successive sensor stations or time windows [15,16], and, in a domestic context, are directly comparable to the deployment reported in this paper—from a deep-learning model trained on signalized-intersection detector data [17]. Collectively, this strand confirms that lane-level resolution is achievable across a range of sensing modalities, but—as with the connected-vehicle literature above—typically as a same-cycle or same-instant estimate rather than the forward-looking, rolling-horizon prediction LQS produces.

A fourth, rapidly growing strand applies computer vision and deep learning directly to camera imagery to detect or estimate queue length, motivated by the same camera-based sensing infrastructure this paper’s own movement-data manager relies on [18,19]; a recent survey situates such models within the broader intelligent-transportation-systems literature and notes that comparatively few have been validated against an independently measured field deployment, a gap this paper’s own field-deployment evidence (Section 2.9 and Section 3.4) is intended to help address for its own case [20].

A fifth strand is the traffic-flow-theory foundation LQS’s downstream arrival-flow model builds on. Robertson’s Platoon Dispersion Model originates from TRANSYT signal-optimization work [21] and remains under active recalibration in the literature for conditions outside its original derivation, including non-lane-based and heterogeneous or mixed traffic streams not considered in this paper [22,23,24]—a body of work methodologically consistent with, though addressing a different model component than, this paper’s own disclosed, site-specific recalibration of the shockwave departure speed (Section 3.3).

A sixth strand, more distant from queue estimation itself, concerns the planning and decision-making for connected and autonomous vehicles at intersections and on multi-lane roadways, and highlights why lane-level queue information of the kind LQS produces is a useful input in the first place: a recent review organized intersection decision-making for connected and autonomous vehicles into graph-based, prediction-based, optimization-based, and learning-based approaches [25], while dedicated work on multi-lane lane-changing decision rules illustrates the kind of downstream consumer this paper’s own message-protocol extension (Section 2.3) is designed to feed [26].

Finally, this paper builds on established C-ITS message-standard work—the SAE J2735 Dedicated Short Range Communications Message Set Dictionary—its Traveler Information Message, in particular [27]—and on our own prior, already-published work on the movement-traffic-volume counting system that supplies LQS’s upstream input data [28]; we treat both as established building blocks rather than re-deriving them here.

Positioned against this now-substantial literature, LQS’s contribution remains the combination of three elements, rather than any one of them in isolation: lane-level, rather than approach- or corridor-level resolution; rolling-horizon short-term prediction rather than a same-instant or same-cycle estimate; and a complete, standards-based real-time system architecture deployed and field-tested outside simulation, rather than an estimation algorithm evaluated only against held-out data.

### 1.3. Contributions

This paper presents four contributions: (i) a rolling-horizon, lane-level queue-length prediction algorithm combining Robertson’s Platoon Dispersion Model and shockwave theory; (ii) a real-time system architecture that integrates this algorithm into a live C-ITS stack via a standards-based message extension, deployed at an operating C-ITS/autonomous-driving testbed, including a field-measured processing-latency result for the deployed message pipeline; (iii) a quantitative validation of the prediction algorithm on an independent microsimulation testbed, with calibration and evaluation on separate signal cycles, a comparison against baseline methods that use the same inputs, a full-time-series error and correlation analysis, and a calibration sensitivity analysis; and (iv) a transparent account of the system’s limitations (Section 4.5), including a disclosed site mismatch between the validation and field-deployment testbeds and the absence of a field measurement of queue-prediction accuracy at the deployment site.

## 2. Methods

### 2.1. System Architecture Overview

**Figure 1:**
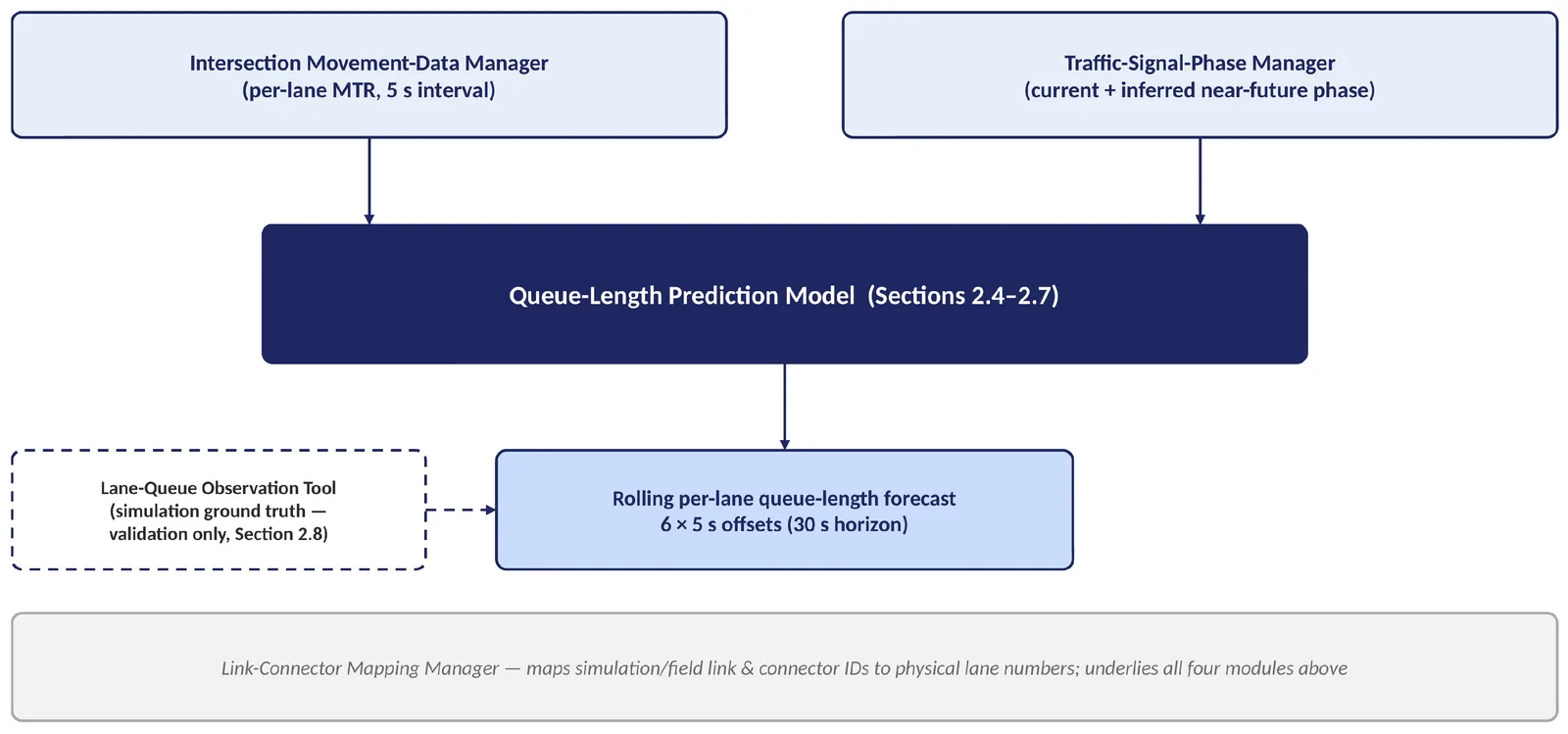
LQS system architecture: five cooperating sub-modules. The link-connector mapping manager is a static lookup table internal to LQS, which the other modules query by link and lane identifier. MTR, movement-traffic-volume record.

LQS is organized as five cooperating sub-modules (Figure 1). The first, a link-connector mapping manager, is an internal component of LQS: a static lookup table, loaded once at initialization, that records per intersection the correspondence between simulation- or field-assigned link/connector identifiers and physical lane numbers, together with lane storage lengths and lane-to-signal-group assignments. Because a queue that spans multiple road segments must be tracked as a single continuous entity across those segments, the other four sub-modules resolve every link and lane identifier through this table. It exposes no runtime interface and is not modified during operation; since the mapping depends only on road geometry and lane configuration, it is updated offline when the intersection layout or lane assignment changes. An intersection movement-data manager collects, per unit time interval (5 s in the current deployment), the movement-traffic-volume record (MTR) for each entry-exit lane pair at each monitored intersection—this sub-module is the one published separately as a movement-traffic-volume counting system [28]; LQS consumes its output as an input feed rather than re-implementing it. A lane-queue observation tool is used during model development and simulation-based validation to extract ground-truth queue length directly from the microsimulation (Section 2.8); in the field deployment, no equivalent ground-truth observation exists in real time, since real-time queue length is precisely what LQS itself is predicting. A traffic-signal-phase manager ingests the current and (via one-cycle history) inferred near-future signal phase per lane. Finally, the queue-length prediction model (Section 2.4, Section 2.5, Section 2.6 and Section 2.7) consumes the outputs of the other four modules to produce the system’s core output: a rolling, per-lane, near-future queue-length forecast.

### 2.2. Data Flow and Field Interfaces

**Figure 2:**
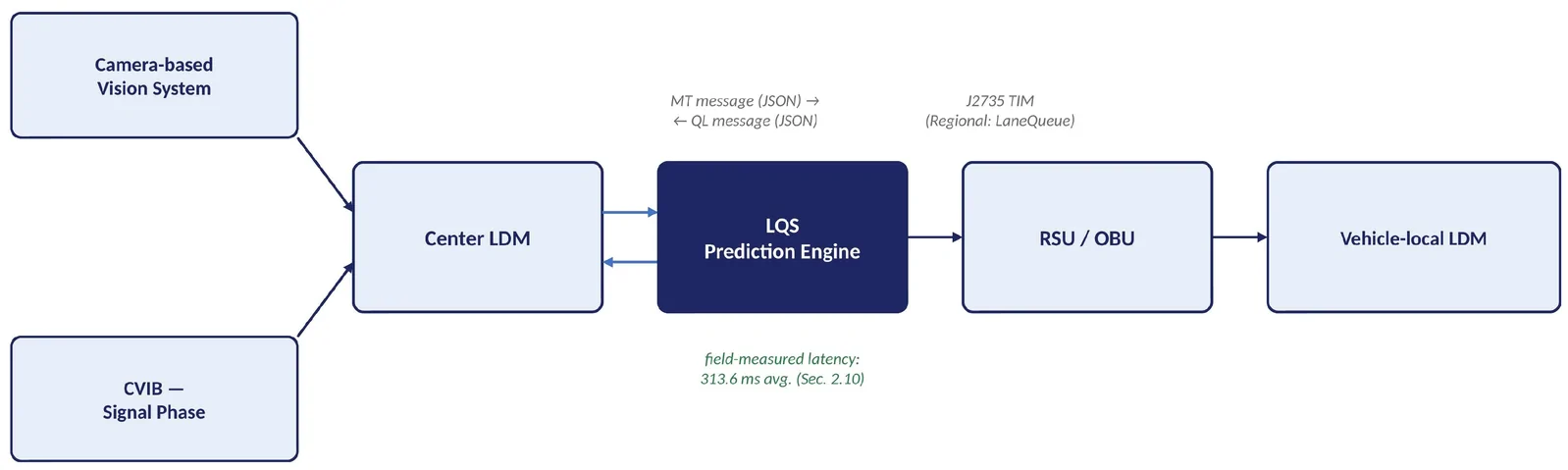
Field-deployment data flow and interfaces, from camera- and signal-phase input through LQS to the connected vehicle.

In the field deployment, LQS sits between two upstream data sources and one downstream consumer (Figure 2). Movement-object detection is provided by a camera-based vision system, and signal-phase data by a Connected Vehicle Interface Board (CVIB) system. Both feeds arrive at a Center Local Dynamic Map (LDM) system, which in turn relays a combined precision-map, moving-object, and signal-phase payload to LQS as a JSON-encoded “MT message” over a negotiated-period TCP socket connection, with the Center LDM acting as the TCP server and LQS as the client. The connection is persistent. At the transport layer, TCP provides ordered delivery and retransmission, so a lost packet reaches LQS as additional delay rather than as a missing or reordered MT message. At the application layer, however, the deployed implementation includes no explicit handling of delayed messages or connection loss: there is no timeout on incoming MT messages, no rule for when buffered input becomes stale, and no automatic re-establishment of a dropped session (Section 4.5). LQS returns its predictions to the Center LDM as a JSON-encoded “QL message” (time-indexed, per-lane predicted queue length), which the deployment specification requires to be delivered within 1000 ms of receipt of the triggering MT message—a real-time latency budget the system must meet to be useful for CAV lane-management decisions made on a timescale of seconds. Section 2.9 reports a field measurement of this processing latency against the deployed system, distinct from the 1000 ms figure stated here as the design requirement. From the Center LDM, lane-queue data is further packaged for vehicle consumption as a J2735 TIM message (Section 2.3) and relayed to onboard units via roadside units (RSU/OBU) and, on the vehicle side, a vehicle-local LDM, per the deployment’s engineering specification.

### 2.3. Message-Protocol Extension

**Figure 3:**
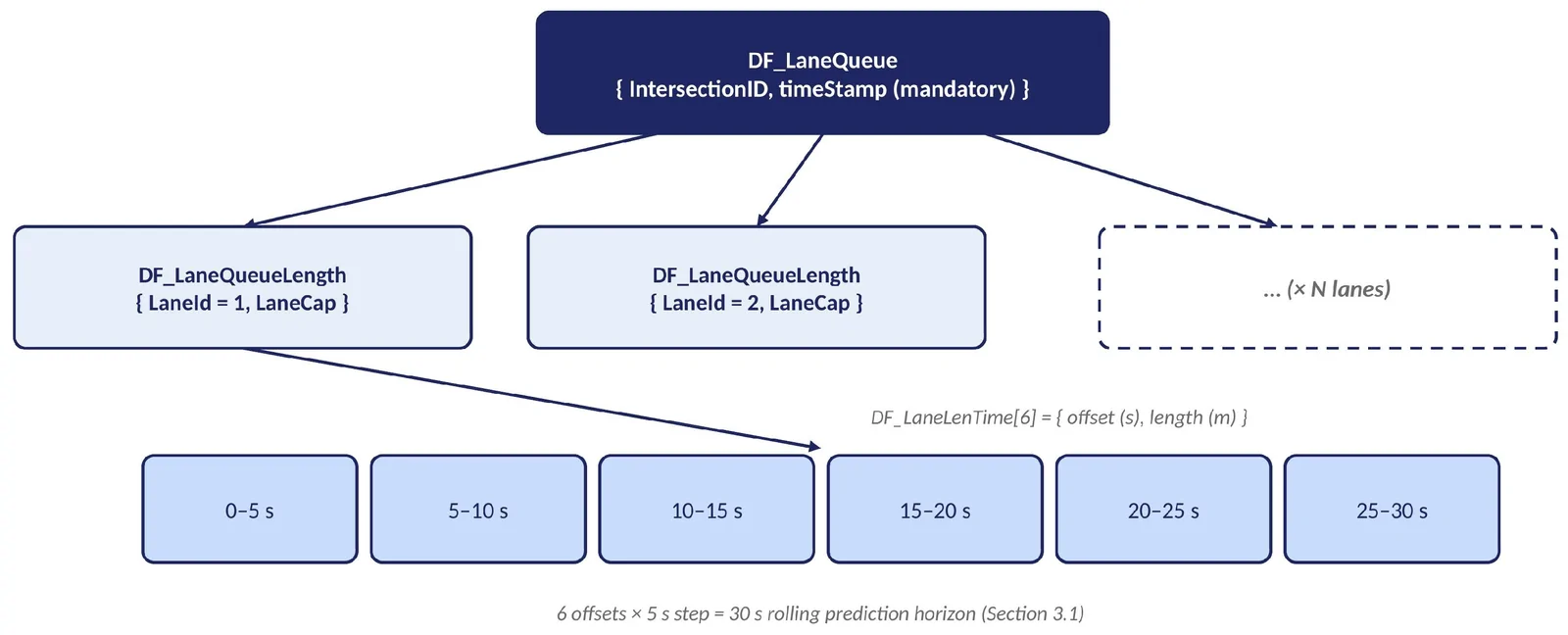
Regional TIM extension: DF_LaneQueue data-frame structure.

Lane-level queue-length data is carried as a regional-part extension to the standard J2735 Traveler Information Message (TIM) rather than as a new message type, with the reasoning that a vehicle consuming lane-queue data has, by construction, already received the corresponding MAP message describing the intersection’s lane geometry. One TIM message instance covers exactly one intersection, meaning a vehicle receiving queue predictions for multiple intersections ahead of it must receive one TIM message per intersection. The extension is structured as a data frame DF_LaneQueue (Figure 3), containing an intersection identifier (IntersectionID, per the base J2735 specification) and a list of per-lane entries DF_LaneQueueLength, each carrying a lane identifier (LaneId), an optional lane capacity (LaneCap, the physical maximum queue length in meters), and a list of predicted-queue-length-at-offset entries (DF_LaneLenTime), each an (offset-in-seconds, queue-length-in-meters) pair—six such offset entries in the current deployment, corresponding to a 5 s-step, 30 s rolling prediction horizon. One design decision is worth noting explicitly: the base TIM specification leaves timeStamp optional, but the lane-queue extension makes it mandatory, since a queue-length prediction is only meaningful relative to a specific reference time.

### 2.4. Problem Formulation

LQS predicts queue length on a rolling-horizon basis: given movement-traffic-volume input aggregated over 5 s timesteps, the system produces, at every timestep, a forecast of per-lane queue length at each of the next six 5 s offsets (a 30 s prediction window). The window length is bounded above by the average travel time across the link being predicted, since queue-length prediction cannot meaningfully extend past the point in which the downstream-arrival-flow prediction itself (Section 2.5) becomes undefined in the current deployment this bound is realized as a fixed 30 s window. The prediction repeats at every timestep as new movement-traffic data arrives, so the system is continuously re-forecasting rather than committing to a single forecast per signal cycle. Unless stated otherwise, the accuracy figures in Section 3 evaluate, for each timestep, the most recent forecast of that timestep (the zero-offset value of the forecast issued at that timestep); Section 3.2 reports accuracy separately for each forecast offset.

### 2.5. Downstream Arrival-Flow Prediction

Upstream movement-traffic volume—the all-lane count of vehicles entering the link under study in each 5 s timestep—was converted to a predicted per-lane downstream arrival-flow profile using the recursive form of Robertson’s Platoon Dispersion Model [21]: q_l(t + τ) = F·p_l·q_u(t) + (1 − F)·q_l(t + τ − Δt), where q_u(t) is the upstream count, p_l is the share of lane l (Section 2.6), q_l is the predicted arrival flow at the stop-line end of lane l, Δt is the timestep (5 s), and τ is the mean link travel time T = L/$\bar{v}$ (L = 450 m, $\bar{v}$ = 60 km/h, T = 27 s) truncated to a whole number of timesteps (τ = 25 s). The smoothing factor is F = 1/(1 + αT). Robertson’s original formulation uses F = 1/(1 + αβT) with a travel-time factor β, and the TRANSYT default values are α = 0.35 and β = 0.8; the implementation validated here omits β (equivalently, β = 1) and uses α = 0.01 s^−1^, which gives F = 0.787 and therefore a weak dispersion. This value was chosen during model development on the same testbed rather than estimated from observed platoons, and it is fixed: it does not adapt to traffic composition or to changes in platoon structure. Platoons created by upstream signal coordination are nevertheless represented, because the model’s input is the observed upstream count at 5 s resolution; α governs only how much those platoons spread over the link. The validation traffic is lane-disciplined passenger-car traffic, so the effect of heavy vehicles or non-lane-based mixed traffic on dispersion, for which recalibrated parameters have been reported [22,23,24], is not examined here. Section 4.3 reports the sensitivity of the results to α, including the TRANSYT default.

### 2.6. Lane-Flow-Ratio Prediction

The arrival flow must be split across individual lanes before a per-lane queue model could be applied. LQS used a “myopic” estimate: the share of stop-line departures observed in each lane during the most recently completed signal cycle, taken from the downstream intersection’s movement counts and applied unchanged to upstream arrivals during the current cycle. The share was recomputed once per cycle transition, using 160 s windows aligned with the signal cycle. No correction for demand change between cycles was applied, so the method assumed that lane choice was stable from one cycle to the next. We tested this assumption on the validation data (cycle windows 3–22): the through-lane shares were stable (coefficient of variation 8–9%), whereas the left-turn share (42%) and the right-turn share (32%) varied substantially between cycles, so the assumption held for the through lanes but was weak for the turning lanes. The estimate was further adjusted by an empirically calibrated correction that moved 40% of the right-turn lane’s share to lanes 1–4 with fixed weights of 0.125, 0.325, 0.325, and 0.225 (Section 3.3); this correction was an empirical adjustment rather than a theoretically derived one.

### 2.7. Lane-Level Queue-Length Model

**Figure 4:**
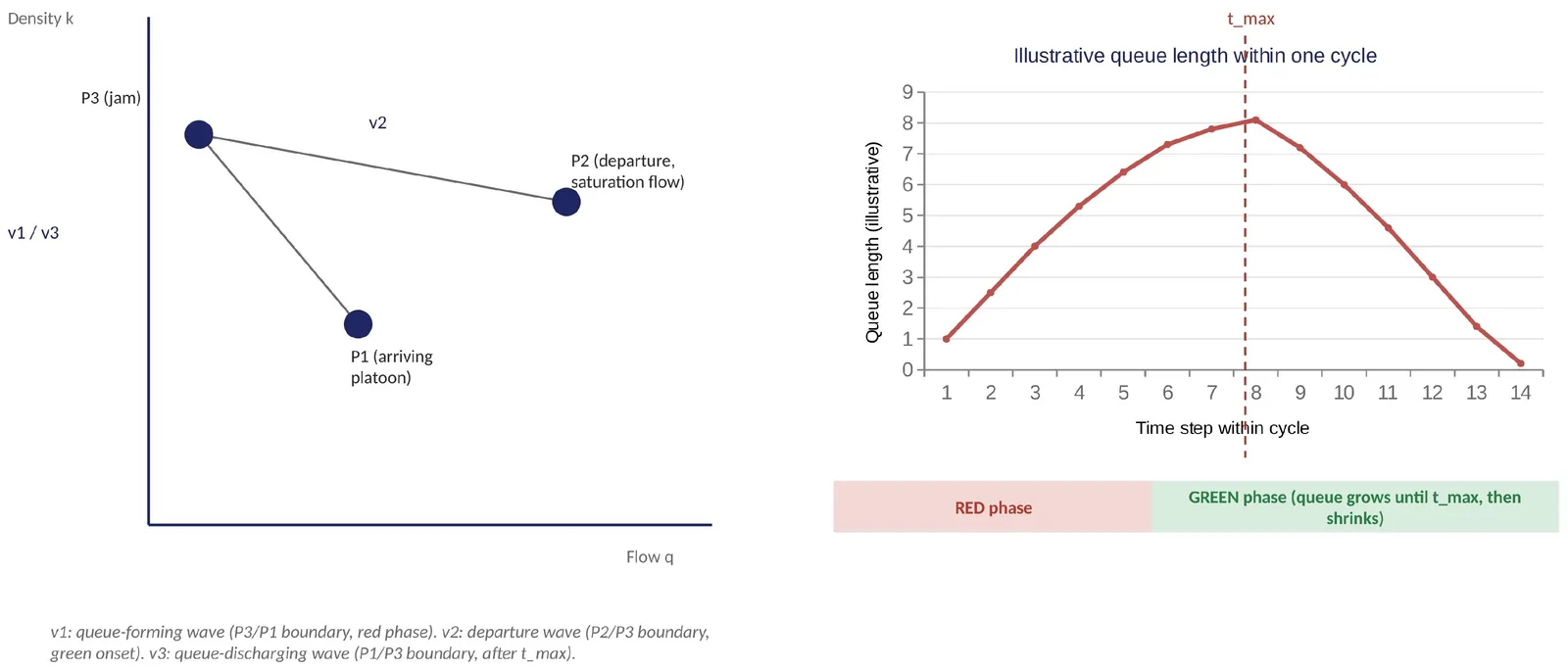
Shockwave-theory schematic: flow-density states (P1, P2, P3) and an illustrative predicted queue-length curve marking t_max.

Given a per-lane, time-dependent downstream arrival-flow forecast (Section 2.5 and Section 2.6) and the corresponding lane’s signal phase, LQS predicts queue-length evolution using a shockwave-theory formulation (Figure 4). Three traffic states were defined on the flow-density diagram: P1, the state of the arriving vehicle platoon approaching the queue; P2, the state of vehicles departing through the intersection under the (uncongested-downstream) assumption that departing traffic can clear at the lane’s saturation flow rate; and P3, the stationary, jam-density state of vehicles within the queue itself. Three corresponding shockwave speeds were defined: v1, the queue-forming wave speed (the boundary between P3 and P1, governing queue growth); v2, the departure wave speed (the boundary between P2 and P3, governing how quickly the front of a green-phase departure wave propagates); and v3, the queue-discharging wave speed (the boundary between P1 and P3, governing queue shrinkage once discharge outpaces arrival).

During a red phase, queue length grows at the v1 wave speed as arriving flow joins the standing queue. At the onset of the green phase, the situation is more subtle than immediate queue dissipation: due to driver perception-reaction time, vehicles at the front of the queue begin moving before vehicles further back, so for a period after the signal turns green, the queue can continue to grow even as its front is discharging—governed by v1 at the rear and v2 at the front—until the discharge wave (v2) overtakes the growth wave (v1) at a critical time we denote t_max. Only after t_max does the queue’s overall length begin to shrink, at rate v3. LQS locates t_max separately per lane at every timestep during a green phase by tracking two auxiliary quantities—a hypothetical queue length resulting from uninterrupted v1-driven growth, and the position of the discharge front resulting from v2-driven propagation since the start of the current green—and treats their crossing as the t_max event (Algorithm 1). If the green phase ends before the two quantities cross, which can occur under short greens or high arrival rates, the queue is not discharged in that cycle: it continues to grow at v1 through the following red, and the front position is reset at the start of the next green. Because nothing in the base model then limited queue growth, LQS capped the predicted queue at the lane’s storage length (60 m for the left-turn pocket and 450 m for the other lanes). Densities feeding into v1 and v3 were computed from the predicted arrival flow and free-flow speed using the fundamental flow–density–speed relationship; in the validation the predicted density never exceeded 30.3% of jam density, so the v1 and v3 expressions remained well behaved without further safeguards. The v2 departure-wave speed, in contrast, was not derived from an arrival-flow-dependent formula but was treated as a per-lane calibration parameter (Section 3.3).
**Algorithm 1.** Per-lane queue update for the forecast issued at timestep t. Notation: Q, predicted queue length; Q_1_, hypothetical v1-driven queue; X, discharge-front position; s, saturation flow (2200 veh/h/lane); ρ_j_, jam density (1/5.1 m); ρ_m_ = ρ_j_/2; S, lane storage length; g, elapsed green time at t, in timesteps.1: for each forecast offset k = 0, …, 5 and each lane l, with predicted arrival flow q = q_l(t + kΔt): 2: ρ ← (q/Δt)/$\bar{v}$; v1 ← (q/Δt)/(ρⱼ − ρ); v3 ← −(s − q/Δt)/(ρₘ − ρ) 3: if k = 0: X ← g·v2·Δt; crossed ← false 4: if the signal of lane l is red: Q ← Q + v1·Δt; Q_1_ ← Q; if the previous step was green: X ← 0, crossed ← false 5: else (green): if not crossed: Q_1_ ← Q + v1·Δt; X ← X + v2·Δt; crossed ← (X ≥ Q_1_) 6:  if crossed: Q ← max(Q + v3·Δt, 0) (after t_max: queue discharging) 7:  else: Q ← Q + v1·Δt (before t_max: queue still growing) 8: Q ← min(Q, S)

### 2.8. Validation Testbed

The prediction algorithm (Section 2.5, Section 2.6 and Section 2.7) was validated on a VISSIM microsimulation testbed of five signalized intersections along a corridor in the Daehwa-dong area of Ilsan-seogu, Goyang-si, Republic of Korea, independent of the Hwaseong field-deployment site described in Section 2.9 (a distinction we return to explicitly in Section 4.1). Simulated demand was calibrated to a level-of-service C–D range per the Korea Highway Capacity Manual [29], with directional-split ratios at each network entry point randomized across 5:5, 6:4, and 3:7 scenarios during calibration, and car-following safety-distance parameters tuned so that simulated intersection capacity matched the Manual’s reference value of 1800 vehicles/hour. The validation reported below used one hour of simulation at 5 s timestep resolution (721 timesteps) on a single representative approach: the northbound approach at the second intersection of the corridor—a five-lane approach comprising one left-turn pocket (lane 1), three through lanes (lanes 2–4), and one right-turn-only lane (lane 5), operating on a 160 s signal cycle (approximately 22–23 complete cycles within the one-hour window). Cycles 1–5 cover the simulation warm-up. Every tuned parameter was calibrated on cycles 6–15, and accuracy was reported on the held-out cycles 16–21. Right turns from lane 5 were permitted on red, as at a real intersection, and during the through green they yielded to pedestrians on the parallel crosswalk; consistent with this, 40.8% of lane 5’s departures in the validation data occured during the through red (none for the through lanes), and its departure rate during green (about 470 veh/h) is far below the saturation flow rate. Lane 5’s discharge is therefore only partly governed by its signal.

### 2.9. Field Deployment Site

LQS was deployed at a C-ITS and autonomous-driving demonstration testbed in the Saesol-dong district of Hwaseong, Republic of Korea, instrumented with cameras across four signalized intersections and covering six approach legs targeted for queue prediction. Camera counts per intersection ranged from two to eight, reflecting differences in the number of approaches and lanes requiring coverage at each location. The LQS server ran on a single rack-mounted server (Intel Xeon Scalable Gold 6128 processor, 3.4 GHz, six cores) under Ubuntu Linux. Field demonstrations conducted on this testbed included both a technical-verification phase (approximately 120 test runs across six days) and a public demonstration phase, during which an illustrative CAV use-case scenario was exercised: an ego vehicle receiving LQS-predicted queue-length information for its current and an adjacent lane, and executing a lane change to the adjacent lane in response to a queue forming ahead of it in its current lane. No quantitative measurement (for example, of travel-time savings or successful-maneuver rate) from this or other field demonstration scenarios is available to report; Section 3.5 treats the field deployment strictly as evidence of real-time architectural feasibility, not as a source of accuracy or benefit figures.

**Figure 5:**
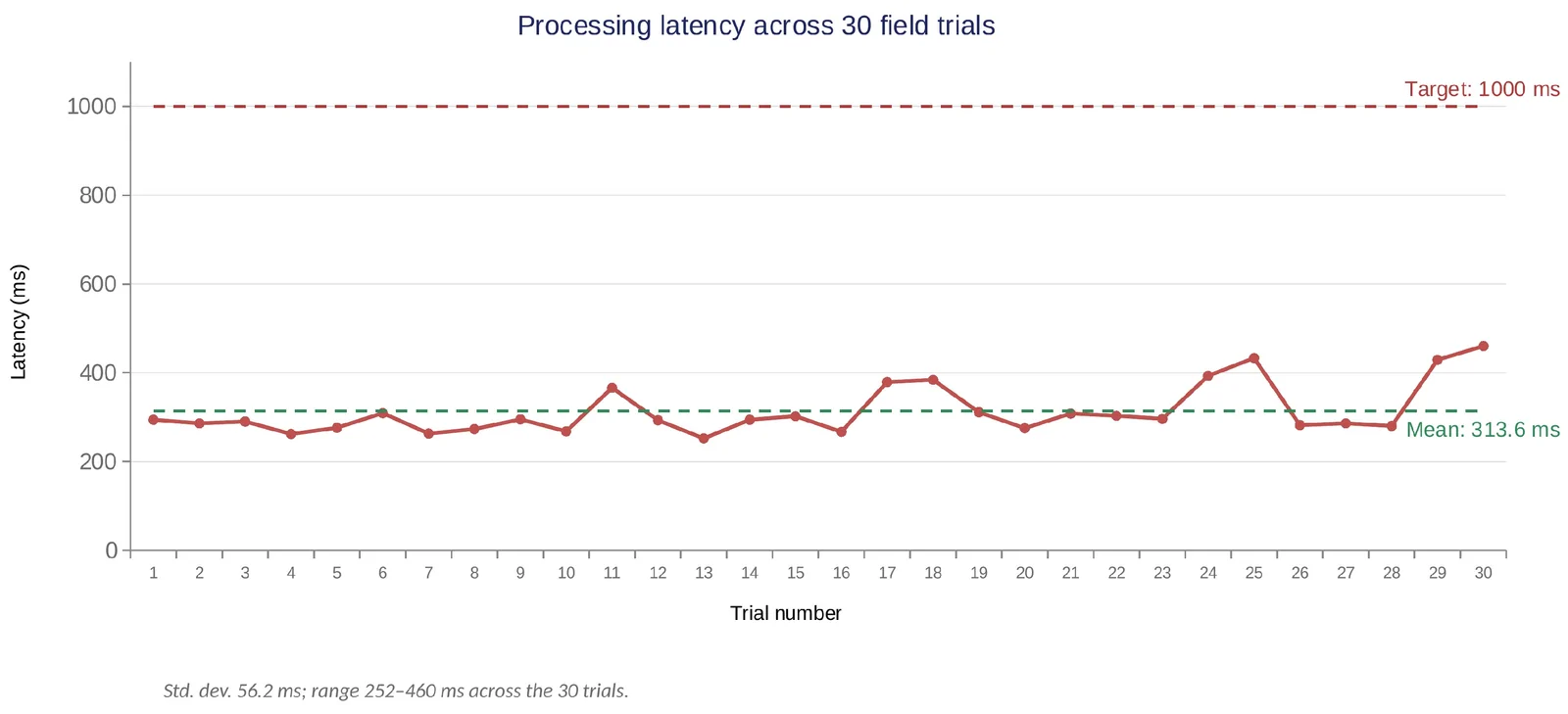
Field processing-latency measurement, 30 trials, against the 1000 ms design budget.

One quantitative field measurement is available, however, and we report it precisely because it is the exception to that statement rather than the rule. A processing-latency verification was conducted against the deployed LQS-to-Center-LDM pipeline: a movement-traffic (MT) message describing traffic moving from approach 1 to approach 3 of the upstream intersection was generated and transmitted to the LQS system from the Center LDM, and LQS’s resulting queue-length prediction for approach 2 of the downstream intersection was generated and returned as a QL message, with an internal LQS logging tool recording the elapsed time between MT-message receipt and QL-message transmission. Across 30 such trials, the measured processing latency averaged 313.6 ms (standard deviation 56.2 ms; range 252–460 ms), well within the 1000 ms design budget stated in Section 2.2 (Figure 5, Section 3.5). The MT message used had been generated for the purpose of this test rather than sourced from live camera-based movement detection, so this is a measured processing-latency result for the deployed system and message pipeline under a controlled, single-movement test input, not a measurement of latency under live, camera-sourced, multi-intersection field traffic.

We report this deployment evidence in three explicit tiers, a distinction Section 3.5 develops further because it directly bears on what this paper can and cannot claim about the Saesol-dong site. Confirmed and quantitatively measured: the processing latency of the deployed MT-to-QL message pipeline, per the test just described (313.6 ms average against a 1000 ms budget). Confirmed by documentation but not independently quantified: the system architecture described in Section 2, the physical infrastructure listed above, and the existence of a live message pipeline connecting the components named in Section 2.2. Documented but not independently measured: the schedule and count of the test runs (approximately 120 over six days) and the occurrence of a public demonstration phase. Not available at any tier: queue-prediction accuracy at this site, and a measured outcome from the ego-vehicle lane-change scenario.

## 3. Results

### 3.1. Validation Accuracy (Cycle-Maximum Relative Error)

We report accuracy as a cycle-level relative error (RE): for each signal cycle, the maximum observed and maximum predicted queue lengths are identified, their relative error is computed, and the result is averaged across cycles. All tuned parameters were calibrated on cycles 6–15, after the simulation warm-up, and accuracy is reported on the held-out cycles 16–21 (Table 1). The calibrated departure-wave speeds are v2 = 4.4, 5.0, 5.1, 8.9, and 4.8 m/s for lanes 1–5. We summarize accuracy by the mean over the four signal-controlled lanes (lanes 1–4) and report the right-turn lane separately, because its discharge is only partly governed by the signal (Section 2.8).

Errors on the four signal-controlled lanes are similar in calibration and validation (mean 25.6% and 24.5%), so the calibrated v2 values do not appear to be over-fitted to cycles 6–15. The right-turn lane deteriorates from 35.3% to 51.5% on the validation cycles, in which its observed queue is very short (mean 1.8 m), so small absolute errors produce large relative errors. The signal-based shockwave model does not represent right turns on red or pedestrian conflicts, and we regard this lane as outside the model’s scope (Section 4.5).

An earlier implementation of LQS, used in the system’s original engineering evaluation, computed the lane shares of Section 2.6 from the signal cycle that was just beginning rather than from the most recently completed one. Those shares are not available in real time, so that configuration acts as an oracle with perfect knowledge of the current cycle’s lane use; we report it as a reference (Section 3.4) rather than as a result. Retaining its parameters while switching to the causal previous-cycle shares makes the predicted queue diverge, because t_max is then not reached in 20 of 22 greens on lane 1 and in 7 of 22 greens on lanes 3 and 4 (Section 2.7); the storage-length cap and the re-calibrated v2 values reported here remove this divergence.

### 3.2. Full-Time-Series Error (RMSE)

The cycle-maximum relative error in Section 3.1 evaluates accuracy at a single moment per cycle—the queue’s peak—and can therefore mask or exaggerate performance at other points in the cycle. To give a fuller picture, we additionally computed the root-mean-square error (RMSE) between predicted and observed queue length over every 5 s timestep of the validation period (cycles 16–21), per lane (Table 2). Forecast offset has little effect: over the validation period, the RMSE of forecasts issued 5–25 s ahead differs from the zero-offset RMSE by at most 2.0 m on any lane, and on lanes 1–4 it is never larger.

As in the cycle-level results, the metrics rank the lanes differently: lane 4 has the lowest validation RE (17.05%) but the largest RMSE (78.28 m), while lane 5 has the largest RE (51.47%) but the smallest RMSE (14.63 m). Section 4.2 shows that this divergence is a consequence of queue scale and is resolved by normalized metrics.

### 3.3. Calibration Sensitivity Analysis

The algorithm as validated above includes two empirically calibrated adjustments beyond the base model described in Section 2: (a) the lane-flow-ratio correction described in Section 2.6, which redistributes 40% of the right-turn lane’s estimated arrival share to the other four lanes using fixed weights; and (b) a per-lane departure shockwave speed (v2, Section 2.7) set to calibrated values rather than the base flow-density value (6.23 m/s). We quantify their individual and joint contribution via a controlled ablation, re-running the validation with each adjustment switched on or off; wherever v2 is tuned, it is re-calibrated on cycles 6–15 for that configuration (Table 3).

Two findings follow from this table. First, each adjustment, applied in isolation, meaningfully changes accuracy relative to the unadjusted baseline, confirming that both are doing real work. Second, the two adjustments are coupled. With the base v2, lane 4’s predicted queue diverges on the validation cycles whether or not the correction is applied (114.0% RE), and the correction alone improves lanes 2, 3, and 5 but worsens lane 4 on the calibration cycles (26.7% to 55.6%). Re-tuning v2 alone repairs lane 4 (16.3%/18.9%) but leaves the right-turn lane poorly predicted (65.3%/136.3%). Only a combination performs well on every lane in both periods. We take this as the paper’s central methodological caveat regarding the algorithm’s calibration: the two adjustments should be understood as a jointly tuned pair specific to this testbed’s demand and geometry, not as two independently justified corrections, and a reader applying this model to a different site should expect to re-tune both together rather than either alone.

### 3.4. Comparison with Baseline Methods

To assess the contribution of each component, we compared LQS with variants and simpler methods that use the same inputs (upstream counts and signal phase), each calibrated on cycles 6–15 by the same procedure and evaluated on cycles 16–21 (Table 4). B1 removes platoon dispersion (F = 1, a pure travel-time shift). B2 splits arrivals equally across lanes, and B3 uses the running mean of the lane shares of all completed cycles instead of the most recent cycle; both re-tune v2. B4 replaces the shockwave queue model with a deterministic point-queue (D/D/1) model on the same predicted lane arrivals, discharging at the saturation flow rate after a start-up lost time calibrated to 5 s, with queue length taken as the number of queued vehicles times the jam spacing (5.1 m). The oracle row repeats the original engineering configuration, which knows the current cycle’s lane shares (Section 3.1). Corridor-level and probe-vehicle methods [4,9,10,11] were not re-implemented: they require high-resolution detector events or vehicle trajectories, which the movement-count interface of Section 2.2 does not provide, so on this testbed they would not operate on comparable inputs.

LQS has the lowest mean RE on the signal-controlled lanes (24.5%), against 33.2% for the running-mean lane share (B3), 34.3% for the point-queue model (B4), and 52.8% for an equal split (B2). By full-series RMSE the differences are small, and B3 is slightly lower (56.4 m versus 59.1 m), so the advantage of LQS lies mainly in the cycle-peak queue that RE measures. Removing dispersion (B1) leaves the result unchanged (24.5%), showing that at the calibrated α the dispersion model contributes almost nothing on this testbed (Section 4.3). Finally, the causal model matches the oracle on lanes 1–4 (24.5% versus 24.5%): on the signal-controlled lanes, the previous cycle’s lane shares carry nearly all the information that perfect knowledge of the current cycle would provide, and the oracle’s advantage on the calibration cycles (22.7% versus 25.6%) does not carry over to the validation cycles.

### 3.5. Field Deployment: Scope of Evidence

The Hwaseong Saesol-dong deployment described in Section 2.9 demonstrates that the architecture in Section 2—camera-based movement input, signal-phase input, the prediction algorithm, and a Center LDM/J2735 TIM message pipeline to connected vehicles—was built and operated as an integrated system outside of simulation. We present this deployment strictly as evidence of real-time architectural and integration feasibility, not as a source of accuracy figures: no ground-truth queue-length measurement was operated in parallel with LQS at the field site, so there is no field analogue to the validation in Section 3.1, Section 3.2, Section 3.3 and Section 3.4. The ego-vehicle lane-change scenario (Section 2.9) is reported descriptively only. The one field measurement, the processing latency of the deployed MT-to-QL pipeline (313.6 ms average over 30 trials, Figure 5), was obtained with a single, purpose-generated input message rather than live, camera-sourced, multi-intersection traffic.

## 4. Discussion

### 4.1. Site Mismatch Between Validation and Deployment

The quantitative validation in Section 3 was performed on a VISSIM model of a Goyang-si (Daehwa-dong) corridor, while the field deployment in Section 3.5 operates at a physically distinct site in Hwaseong (Saesol-dong). We disclose this mismatch explicitly rather than blurring the two into a single “the system works” narrative. Our position is that this is a legitimate two-part contribution rather than a validation gap masquerading as two separate results: the prediction algorithm of Section 2 is general-purpose traffic-flow theory, not calibrated to any specific intersection geometry beyond the lane-flow-ratio and shockwave-speed parameters discussed in Section 3.3 (which are themselves disclosed as site-specific and not claimed to transfer); a rigorous accuracy validation at one representative site, combined with a separate demonstration of real-time engineering feasibility at another, together support the claim that the algorithm is both accurate (where validated) and deployable (where deployed)—without claiming that the deployed instance’s accuracy at Hwaseong had been measured. A direct re-validation of the algorithm on a VISSIM model of the actual Hwaseong Saesol-dong geometry would close this gap entirely; we did not attempt this here and list it as the highest-priority item in Section 4.6.

### 4.2. Resolving the RMSE/Relative-Error Divergence on Lane 4

Section 3.2 reported that lane 4 simultaneously had the lowest cycle-maximum relative error and the largest full-series RMSE among the five lanes studied. We resolve this divergence directly, by extracting the predicted and observed queue-length series for every lane over the validation period (cycles 16–21) and computing, per lane, the RMSE normalized by that lane’s mean observed queue length, the mean signed bias (predicted minus observed), and the Pearson correlation coefficient between the predicted and observed series (Table 5).

The normalized figures explain the apparent divergence: lane 4’s raw RMSE is large in absolute terms only because lane 4 carries the largest mean queue of the five lanes (97.6 m, versus 1.8 m for lane 5); once RMSE is expressed relative to that mean queue, lane 4’s normalized error (80.2%) lies in the same range as lanes 1–3 (73.6–88.2%), consistent with its low cycle-maximum relative error. Lane 5, by contrast, has a small absolute RMSE only because its queues are very short; normalized against its own mean queue, its error (814.0%) is by far the largest, and its correlation (0.04) shows that the model does not track this lane’s queue. Taken together, the raw and normalized metrics agree that lanes 1–4 are predicted with similar relative accuracy and that lane 5 is not, and the earlier apparent puzzle was a consequence of comparing absolute errors across lanes with very different queue scales.

The signed bias is negative on the through lanes (lanes 2–4: −12.8, −36.0, and −22.8 m in validation; −16.8, −9.9, and −6.7 m in calibration), i.e., queues on these high-volume lanes are systematically under-predicted. Lane allocation is not the cause: over the hour, the predicted arrivals on lanes 2–4 exceed the observed stop-line departures by 3–6%. Two mechanisms are more plausible. First, the departure-wave speed v2 is calibrated to minimize cycle-maximum RE, not bias; in our tests, lowering v2 on lanes 2–3 reduces the bias but moves the model towards the threshold at which t_max is no longer reached and the queue diverges (Section 4.3). Second, arrivals are shifted by a fixed free-flow travel time (25 s), whereas vehicles approaching a standing queue decelerate, and the queue observed in the simulation includes slow-moving vehicles joining its tail; both make the observed queue longer than the model’s stationary-queue representation during queue growth. We have not separated these two effects quantitatively.

### 4.3. Calibration Transparency and Robustness

Following Section 3.3, the two empirically tuned elements remain in the algorithm and are presented with their sensitivity rather than as fixed constants. Two further sensitivities matter for anyone re-calibrating the model. First, v2 lies close to an instability threshold: lowering all v2 values by 0.3 m/s raises the mean validation RE on lanes 1–4 from 24.5% to 46.0% (and to 73.3% at 0.5 m/s), whereas raising them by 0.3 or 0.5 m/s changes it only to 25.0%. Below the threshold, t_max is not reached within the green and the queue accumulates over successive cycles; without the storage-length cap, the predicted queue in this regime reached 2098 m on a 450 m link. Calibration should therefore favor the upper side of the optimum. Second, the dispersion parameter has little influence at its calibrated value: with v2 re-calibrated each time, the mean validation RE on lanes 1–4 is 24.5% without dispersion (F = 1), 24.5% at α = 0.01 s^−1^ (F = 0.787), 26.4% at α = 0.05 s^−1^ (F = 0.426), and 49.6% at the TRANSYT default (αβ = 0.28 s^−1^, F = 0.117). On this testbed, the platoon dispersion model therefore acts essentially as a travel-time shift, and stronger dispersion degrades the prediction. We expect dispersion to matter more on longer links and in heterogeneous traffic [22,23,24], which the present testbed does not test.

### 4.4. Real-Time and Scalability Considerations

The field deployment’s sub-1000 ms processing-latency requirement was measured, not merely assumed, for the single MT-to-QL prediction pipeline described in Section 2.9: 313.6 ms average over 30 trials. This measurement was taken under a single, controlled test movement between two intersections, not under the full deployed scale (four intersections, six approach legs, up to eight cameras per intersection) processing live, concurrent, camera-sourced traffic. Latency under that load was not measured, but its scaling can be estimated. The prediction for each approach is computed independently, at a cost proportional to the number of lanes times the number of forecast offsets. In an offline benchmark of the validation implementation on a single processor core, the algorithm required about 0.05 ms per 5 s timestep for one five-lane approach and was scaled linearly, to about 0.29 ms for six approaches. The algorithmic share of the measured 313.6 ms is therefore below 0.1%; most of the latency lies in message parsing, map handling, and socket communication, which grew with the size of the MT message, i.e., with the number of cameras and movements reported. We therefore expect latency at full deployment scale to grow approximately linearly with MT-message size rather than with the number of predicted lanes. This is an estimate rather than a measurement, and the field implementation may differ from the offline code; a load test remains as future work (Section 4.6).

### 4.5. Limitations

We collect the paper’s limitations here in one place. (1) The field deployment does not demonstrate field-measured queue-prediction accuracy: one aspect of its real-time performance, processing latency, has a field measurement behind it (313.6 ms average), but no ground-truth queue length was collected in parallel with LQS’s live predictions, so the accuracy claim of Section 3 is a simulation-based claim at a different site (Section 4.1). (2) The validation covers a single approach under one calibrated demand scenario and one simulation run, and the held-out validation period comprises six signal cycles; accuracy across other geometries, lane configurations, and the directional-split scenarios (5:5, 6:4, and 3:7) used during testbed calibration has not been examined. (3) The right-turn lane is outside the model’s scope: right turns on red and pedestrian conflicts during the through green decouple its discharge from the signal, and its prediction error is reported separately rather than included in the summary metric. (4) The calibration parameters are site-specific and coupled (Section 3.3), and v2 lies close to an instability threshold (Section 4.3), so re-calibration at a new site must be done jointly and conservatively. (5) The latency measurement is narrowly scoped: a single test movement between two intersections with a synthetically generated input message. (6) The deployed implementation has no application-level handling of delayed MT messages or connection loss (Section 2.2): no timeout, no staleness rule for buffered input, and no automatic reconnection, so the robustness of LQS to communication faults is untested. (7) The baseline comparison (Section 3.4) is limited to methods that operate on the same movement-count inputs; methods based on detector events or vehicle trajectories were not compared.

### 4.6. Future Work

Future work would follow directly from these limitations: (1) re-validation on a VISSIM model of the Hwaseong, Saesol-dong geometry (Section 4.1), closing the site-mismatch gap directly rather than only disclosing it; (2) a field measurement campaign at Saesol-dong, collecting an independent, synchronized ground-truth queue-length record against LQS’s live predictions, which is the only evidence that would support a field-accuracy claim; (3) validation across the directional-split scenarios (5:5, 6:4, and 3:7), additional approaches, and multiple simulation runs; (4) a dedicated model for right-turn lanes that represents turns on red and pedestrian conflicts; (5) a robust handling of delayed and missing inputs, including stale-data flags in the QL message, degraded-mode prediction during communication gaps, and automatic reconnection; (6) a load test of latency and throughput under concurrent, live, camera-sourced traffic across the full four-intersection deployment (Section 4.4); and (7) a density correction based on Edie’s generalized definitions and an LSTM-based trajectory model, using probe-vehicle data, which was identified during the system’s engineering design but has not been implemented beyond a conceptual stage.

## 5. Conclusions

This paper presents LQS, a real-time lane-level queue-length prediction system combining Robertson’s Platoon Dispersion Model and shockwave theory on a rolling-horizon scheme, together with its integration into a live C-ITS message-standard stack and its deployment at an autonomous-driving testbed. Using only information available at prediction time, with parameters calibrated on signal cycles 6–15 and evaluated on held-out cycles 16–21, LQS achieved a mean cycle-maximum relative error of 24.5% on the four signal-controlled lanes, lower than equal-split, running-mean lane-share, and point-queue baselines and equal to an oracle that knows the current cycle’s lane use. The right-turn lane, which discharges on red and yields to pedestrians during green, lies outside the model’s scope. Normalized RMSE, bias, and correlation reconciled the divergence between cycle-maximum and full-series errors, and identified a systematic under-prediction on the through lanes. A controlled ablation showed that the two calibration parameters were coupled, and a sensitivity analysis showed that the departure-wave speed lied close to an instability threshold and that platoon dispersion contributes little on this testbed—findings with direct implications for re-calibrating the model at a new site. Positioned against prior queue-estimation literature (Section 1.2), LQS’s contribution is a combination of lane-level resolution, rolling-horizon prediction, and a complete, standards-based real-time system deployed outside simulation, rather than any one of these individually. The field deployment demonstrates architectural and integrational feasibility at Hwaseong, Saesol-dong, with one field measurement (313.6 ms average processing latency under a controlled test) but not field-measured queue-prediction accuracy (Section 3.5), which remains the clearest priority for future work (Section 4.6).

## Figures and Tables

**Table 1:** Cycle-maximum relative error (RE) by lane: calibration cycles 6–15 and held-out validation cycles 16–21, VISSIM validation testbed.

Lane	Movement	RE, Calibration (Cycles 6–15)	RE, Validation (Cycles 16–21)
1	Left-turn	43.11%	28.83%
2	Through	21.71%	22.14%
3	Through	23.71%	29.96%
4	Through	13.89%	17.05%
5	Right-turn	35.26%	51.47%
Mean	Lanes 1–4	25.61%	24.49%

**Table 2:** Full-time-series root-mean-square error by lane, validation period (cycles 16–21).

Lane	RMSE (m)
1	16.14
2	64.35
3	77.52
4	78.28
5	14.63

**Table 3:** Calibration ablation: cycle-maximum relative error (%) by lane under four configurations of the two empirically tuned parameters, shown as calibration cycles 6–15/validation cycles 16–21.

Configuration	Lane 1 RE	Lane 2 RE	Lane 3 RE	Lane 4 RE	Lane 5 RE
Neither adjustment	43.2/31.3	27.2/31.1	37.3/34.4	26.7/114.0	76.6/136.3
Tuned v2 only	43.2/32.5	26.7/27.1	29.3/34.2	16.3/18.9	65.3/136.3
Lane-flow-ratio correction only	43.1/27.5	22.9/26.6	30.2/30.0	**55.6/114.0**	35.3/51.1
Both adjustments (as validated)	43.1/28.8	21.7/22.1	23.7/30.0	13.9/17.1	35.3/51.5

**Table 4:** Comparison with baseline methods using the same inputs: cycle-maximum relative error (RE) and RMSE. All parameters were calibrated on cycles 6–15; validation on cycles 16–21.

Model	Mean RE, Lanes 1–4, Calibration (%)	Mean RE, Lanes 1–4, Validation (%)	Max RE, Lanes 1–4, Validation (%)	RE, Lane 5, Validation (%)	Mean RMSE, Lanes 1–4, Validation (m)
LQS (causal, this paper)	25.6	24.5	30.0	51.5	59.1
Oracle reference (current-cycle lane shares)	22.7	24.5	30.7	103.2	60.3
B1: no platoon dispersion	25.4	24.5	29.4	52.9	61.5
B2: equal lane split	61.7	52.8	59.8	306.4	72.1
B3: running-mean lane share	35.8	33.2	41.4	255.5	56.4
B4: point-queue (D/D/1) model	37.0	34.3	41.3	55.0	60.0

**Table 5:** Normalized RMSE, bias, and Pearson correlation by lane, validation period (cycles 16–21).

Lane	RMSE (m)	Mean Observed Queue (m)	Normalized RMSE (%)	Bias (m)	Pearson r
1	16.14	18.30	88.2	−4.46	0.398
2	64.35	87.44	73.6	−12.80	0.534
3	77.52	92.03	84.2	−36.03	0.480
4	78.28	97.61	80.2	−22.77	0.456
5	14.63	1.80	814.0	+6.40	0.037

## Data Availability

The data presented in this study are not publicly available due to security and confidentiality restrictions associated with the government-funded infrastructure research project under which they were collected. Requests to access the data should be directed to the corresponding author, subject to institutional approval.

## Abbreviations

The following abbreviations are used in this manuscript:

LQS	Lane-by-lane Queue Information Prediction System
C-ITS	Cooperative Intelligent Transport System
CAV	Connected and Autonomous Vehicle
TIM	Traveler Information Message
RSU/OBU	Roadside Unit/On-Board Unit
LDM	Local Dynamic Map
CVIB	Connected Vehicle Interface Board
MTR	Movement-Traffic-Volume Record
RE	Relative Error
RMSE	Root-Mean-Square Error
VISSIM	Verkehr In Städten-SIMulationsmodell (microscopic traffic simulation software)
